# Induction therapy with thymoglobulin or interleukin-2 receptor antagonist for Chinese recipients of living donor renal transplantation: a retrospective study

**DOI:** 10.1186/s12882-019-1293-2

**Published:** 2019-03-22

**Authors:** Jiang Qiu, Jun Li, Guodong Chen, Gang Huang, Qian Fu, Changxi Wang, Lizhong Chen

**Affiliations:** Division of Organ Transplantation, the First Affiliated Hospital of Sun Yat-sen University, 58 Zhongshan II Road, Guangzhou, 510080 China

**Keywords:** Living donor renal transplantation, Induction therapy, Rabbit antithymocyte globulin (rATG), Interleukin-2 receptor antagonist (IL2-RA)

## Abstract

**Background:**

Recipients of living donor renal transplantation are typically considered to have a relatively lower immunological risk. This retrospective study aimed to compare the therapeutic efficacy and safety between rabbit antithymocyte globulin (rATG) or interleukin-2 receptor antagonist (IL2-RA) induction therapies in Chinese population.

**Methods:**

A total of 188 patients receiving living donor renal transplantation between February 2004 and December 2013 were included and divided into the rATG group and based on their induction therapy. The primary outcome was clinically-suspected rejection. The incidences of de novo donor-specific antigen (dn-DSA), graft survival, and infection were also compared between groups. A multivariate Cox regression analysis was performed to investigate the influential factors associated with clinically-suspected acute rejection and graft survival.

**Results:**

The rATG group had a higher panel reactive antibody (PRA) score and more complete HLA mismatches than the IL2-RA group (both *P* < 0.001). The incidences of clinically-suspected acute rejection (9.8% vs. 8.8%; *P* = 0.832) and dn-DSA formation (4.9% vs. 5.4%, *P* = 0.44) were not significantly different between groups. Kaplan-Meier curve analysis demonstrated that the graft survivals of two groups were comparable (*P* = 0.857). After adjusting for patients’ age, sex, PRA, HLA mismatch confounders, and the use of corticoids, the multivariate Cox regression analysis showed that methods of induction therapy were not associated with clinically-suspected acute rejection and graft survival (both *P* > 0.05). The incidences of complications (infections, pneumonia, liver injury and myelosuppression) were all comparable between groups (all P > 0.05).

**Conclusions:**

These results suggested that rATG could be a safe and efficient immunosuppressant when used in a Chinese recipient population with a higher immunological risk in living donor renal transplantation.

## Background

Kidney transplantation remains the most optimal therapy for patients with irreversible chronic kidney failure [1]. According to the 2009 KDIGO (Kidney Disease: Improving Global Outcomes) guideline [2], immunosuppressive therapy is crucial for successful kidney transplantation, which purpose is to prevent rejection episodes, maintain the allograft function and minimize the risk of side effects and infection [3]. Immunosuppression regimens typically include a perioperative induction therapy and a life-long maintenance therapy. Induction treatment is capable of effectively reducing the incidence of acute graft rejection, a risk factor for the long-term outcomes of transplantation [4, 5]. Current options for induction therapy mainly include lymphocyte-depleting antibodies (such as polyclonal rabbit antithymocyte globulin [rATG, thymoglobulin]) and monoclonal antibodies against the interleukin-2 receptor (IL2-RA, such as basiliximab and daclizumab) which acts to inhibit T cell proliferation in response to IL-2 [6]. In China, rATG and basiliximab are two of the most commonly used induction therapy regimens.

Recipients of living donor renal transplantation are typically considered to have a relatively lower immunological risk as compared with those of deceased donor renal transplantation [7] due to the better compatibility between donor and recipient, shorter ischemic time and better quality of the graft. Nevertheless, the beneficial effect of induction therapy against acute rejection has also been observed in kidney transplantation with living donor [8]. Compared with rATG, IL2-RA is a less potent immunosuppressant and is recommended as the first-line induction therapy for living donor renal transplantation by the KIDGO guideline to reduce the risk of postoperative immunosuppression-related infections [2]. By contrast, the relatively-more-potent immunosuppressant rATG can reduce the risk of acute rejection as compared with IL2-RA, but also induces higher rates of infection and other side effects [9]. Therefore, rATG is recommended for patients at high risk of graft rejection according to the KIDGO guideline [2]. However, rATG induction has also been shown to be safe and effectively reduce the incidence of acute rejection and complications in living donor renal transplantation [10]. Hence, both rATG and IL2-RA are the options of induction therapy for living donor renal transplantation. Currently, the selection of induction therapy for living donor renal transplantation is commonly based on comprehensive assessment of immunological risk for individual patients. Independent factors associated with increased risk of acute graft rejection include the number of human leukocyte antigen (HLA) mismatches, younger recipient age, older donor age, panel reactive antibody (PRA) score > 0%, presence of a donor-specific antibody (DSA), blood group incompatibility, delayed onset of graft function and cold ischemia time > 24 h [2].

At present, the studies on comparing the safety and efficacy of different induction therapies for living donor renal transplantation are still limited, especially for the Chinese population. Thus, convincing evidence is still lacking to recommend the most appropriate treatment strategies for living donor renal transplantation. Therefore, this retrospective study aimed to compare the therapeutic efficacy and safety between IL2-RA and rATG induction therapies in Chinese recipients of living donor kidney transplantation.

## Methods

### Patients

**Fig. 1:**
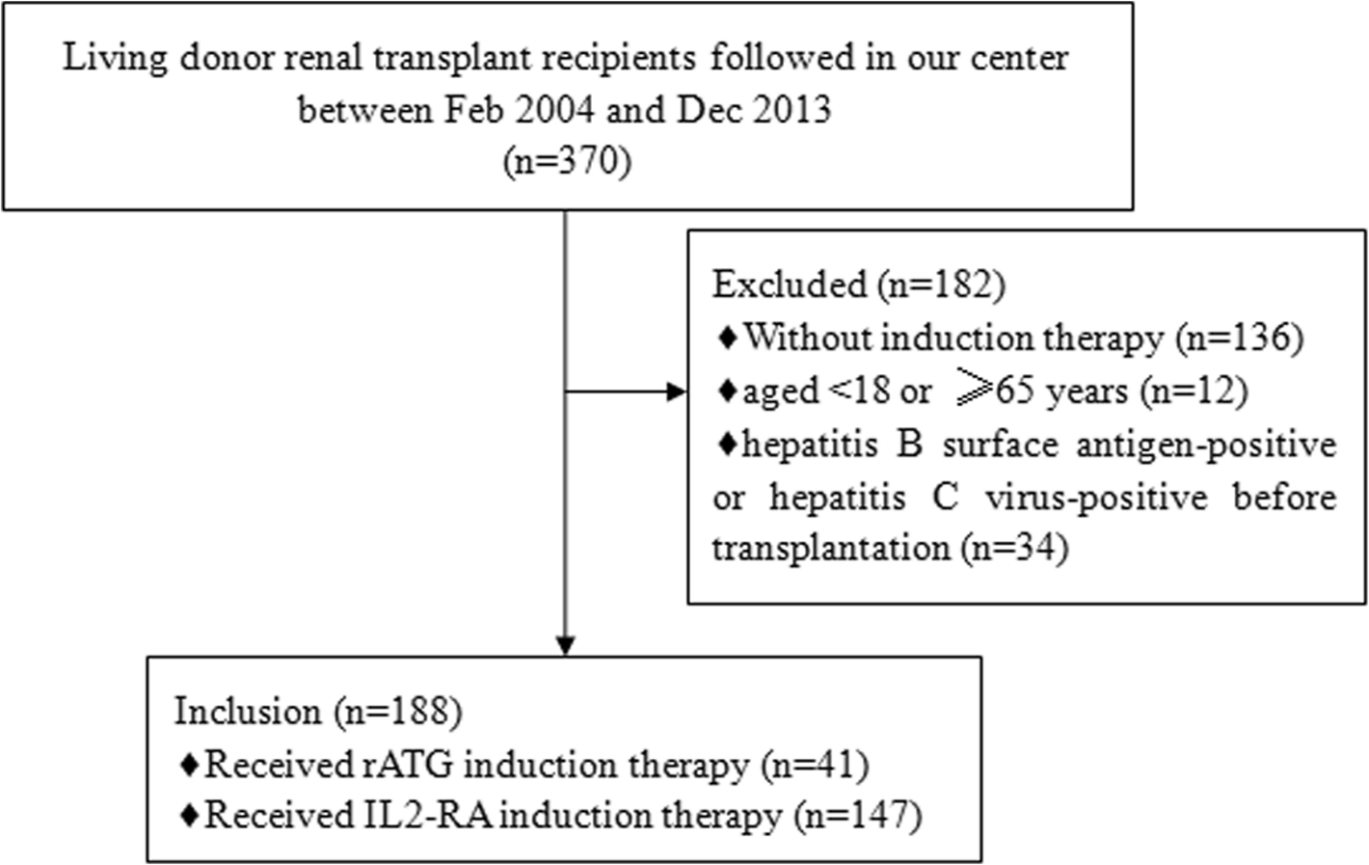
Consort diagram for case selection

A total of 188 patients (age 18–64 years) receiving living donor renal transplantation at our institution between February 2004 and December 2013 were included in this retrospective study. All patients received rATG or IL2-RA as the induction therapy. The exclusion criteria were: 1) received multi-organ transplantation or immunosuppressive therapy before kidney transplantation; 2) hepatitis B surface antigen-positive or hepatitis C virus-positive before transplantation; 3) with cancer within two years prior to kidney transplantation (Fig. 1). This study was approved by the Institutional Review Board of The First Affiliated Hospital of Sun Yat-Sen University, Guangzhou, China. Written informed consent was obtained from each patient.

### Induction therapy

For rATG induction therapy, rATG was intravenously injected at a dose of 1 mg/kg/day from Day 0 to Day 2 post-transplantation. For IL2-RA induction therapy, IL2-RA (basiliximab) was intravenously injected at a dose of 20 mg/day from Day 0 to Day 3 post-transplantation. Methylprednisolone (500 mg/day, intravenously) was given to all patients during the transplantation and on the first two days post-transplantation. The method of induction therapy was determined by the attending physician based on the immunological risk (such as PRA score and mismatch) and infection risk of each patient.

### Maintenance therapy

Patients received MMF 750 mg orally twice daily after kidney transplantation, and the dose was tapered to 500 mg twice daily after 6 months post-transplantation. TAC/cyclosporin was started postoperatively on Day 3. The initial dose of TAC was 0.1 mg/kg/day, and the trough level was 5–10 ng/mL within the first 6 months, and then tapered to 4–6 ng/mL at 1 year and 3–5 ng/mL after 2 years. The initial dose of cyclosporin was 5 mg/kg/day, and the trough level was 150–220 ng/mL within the first year and 150–200 ng/mL after 1 year. Prednisone (30 mg/day) was started postoperatively on Day 3 and tapered to 5 mg/day within 3 months.

All patients received prophylactic intravenous ganciclovir (250 mg/day) against cytomegalovirus (CMV) within the first 2 weeks, followed by oral ganciclovir (3 g/day) for 90 days. Prophylactic sulfamethoxazole plus trimethoprim against *Pneumocystis carinii pneumonia* (PCP) was given orally for 3 months.

### Study outcome measurements

The primary outcome of this study was the incidence of clinically-suspected acute graft rejection within the follow-up period of 80 months, defined as serum creatinine increase > 10% per day and renal arterial resistance index > 0.8. Where possible, patients with suspected acute rejection were confirmed by standard percutaneous kidney allograft biopsy.

Other study outcomes included detection of de novo DSA (dn-DSA) which was carried out for all patients using Luminex (Austin, TX, USA), recipient and graft survival, delayed graft function (defined as requirement for dialysis within the first week after transplantation) and infection. Liver injury was defined as alanine transaminase (ALT) level > 40 U/L or aspartate transaminase (AST) > 37 U/L. Myelosuppression was defined as the leukocyte count (WBC) < 4 × 10^9^/L or the platelet count< 100 × 10^9^/L. The incidence of infection was defined as all the infectious events.

### Management of acute rejection

According to the standard practice in China, following diagnosis of acute rejection, patient was continuously given methylprednisolone (500 mg/day, intravenously) for 3 days. If the rejection was steroid-resistant, then rATG (1 mg/kg/day) was administrated for 7–10 days.

### Statistical methods

Graft survival were analyzed by Kaplan-Meier survival function and compared by log-rank test, and patients lost to follow-up or with missing data were censored. Categorical variables were presented as number and percentage and compared by the χ^2^ test or Fisher’s exact test. Continuous variables were presented as mean ± standard deviation (SD) unless otherwise stated and compared by the Student’s t-test.

Cox proportional hazards regression analysis was performed to investigate the independent factors associated with graft survival or acute rejection. The multivariate Cox regression model was adjusted for confounding factors, including age, sex (male vs. female), PRA (< 10% vs. > 10%), the incidence of complete HLA mismatch (yes or no), the use of corticoids (yes or no) and induction treatment (IL2-RA vs. rATG). For acute rejection analysis, the number of weeks between completion of the transplant and incidence of acute rejection was included in the model. If the date of acute rejection was missing or incomplete, an estimated date of incidence was calculated using creatinine levels. A sudden rise followed by a drop in creatinine level with constant levels of other laboratory values was considered to indicate the time of acute rejection. For patients without an acute rejection, censoring was performed at the date of lost to follow-up. Stepwise regression was used to investigate the strength of covariates in the model.

Propensity score analysis was used to investigate the association between the independent variables and induction therapy groups. Multivariate logistic regression was used to generate the probabilities (propensity score) and these probabilities would be entered into Cox regression models to observe the association between induction therapy and graft survival while the propensity score was adjusted. Multivariate logistic regression was also used to investigate the independent factors associated with clinically suspected acute rejection, graft survival or overall infection.

A *p*-value < 0.05 was considered statistically significant. All analyses were performed by SPSS 16.0 software (SPSS Company, Chicago, IL, USA).

## Results

### Patients

A total of 188 patients (age 18–64 years) receiving living donor renal transplantation were included and divided into rATG group and IL2-RA group based on their induction immunosuppressive therapy. The demographic and clinical characteristics were summarized in Table 1. The age and duration of dialysis were comparable between groups (both *P* > 0.05). Compared to the IL2-RA group, the rATG group had more males, a higher PRA score (30.0% vs. 4.9%, *P* < 0.001), and more cases with complete HLA mismatches (31.7% vs. < 0.68%, *P* < 0.001).

**Table 1:** Comparison of the demographics and clinical characteristics between groups

Variables	rATG (*n* = 41)	IL2-RA (*n* = 147)	*P*-value
Age, years			
Receivers	31.0 ± 12.2	33.5 ± 8.3	0.110
Donors	47.1 ± 9.4	47.2 ± 10.8	0.976
Gender (m/f), n	26/15	123/24	0.005
Dialysis time, months	11.2 ± 8.3	12.2 ± 15.4	0.700
PRA score, %	30.0	4.9	< 0.001
Complete HLA mismatch, n (%)	13 (31.7)	1 (0.68)	< 0.001
Median follow-up time, weeks (range)	45 (17–96)	37 (17–73)	0.540
Renal transplantation history	2 (6.2)	1 (0.8)	0.106

All values are mean ± standard deviation unless specified

HLA = human leukocyte antigen; IL2-RA = IL-2 receptor antagonist; PRA = panel reactive antibody; rATG = rabbit antithymocyte globulin

### Outcomes of renal transplantation

**Fig. 2:**
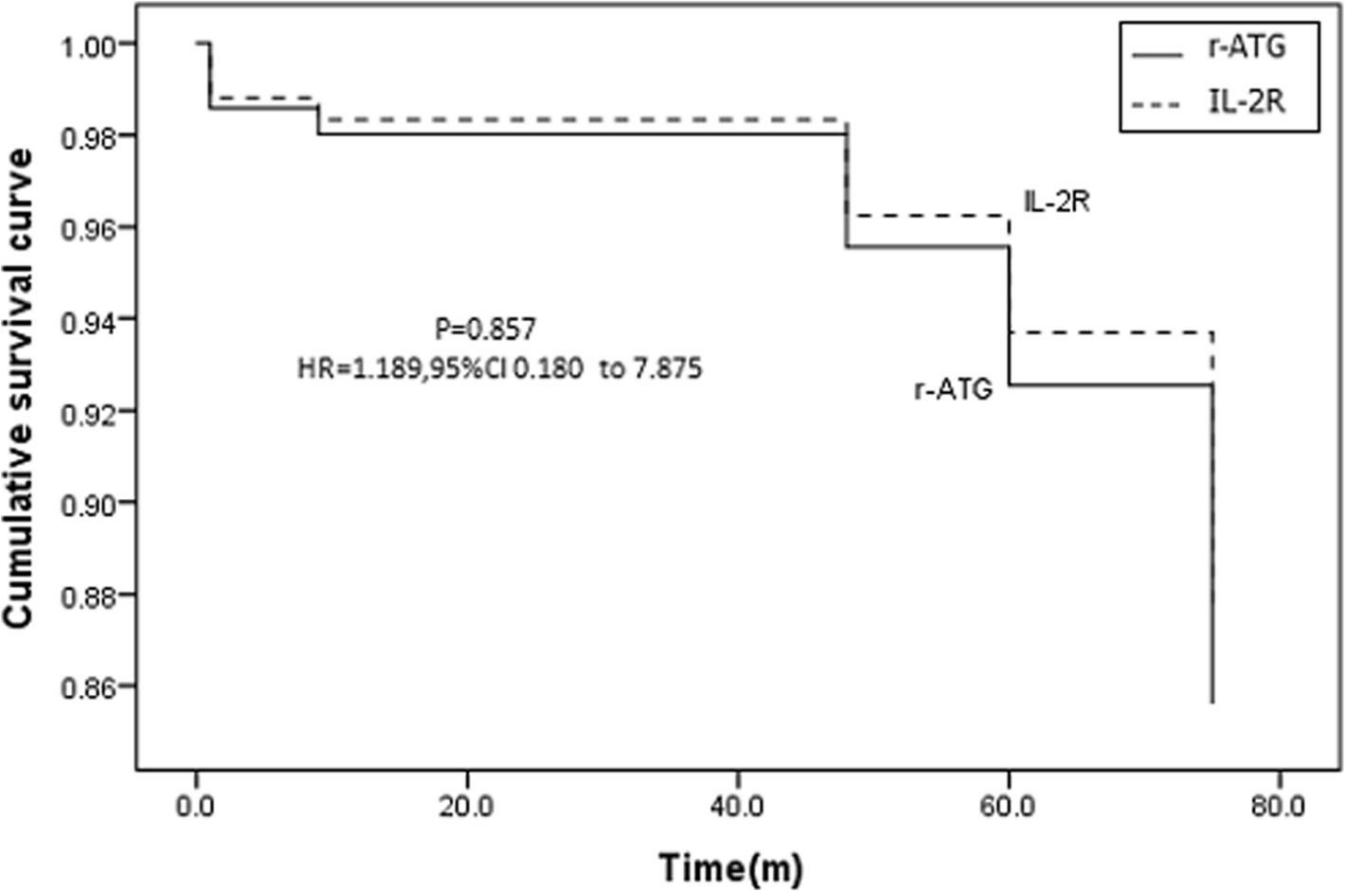
Graft survival rate for renal transplant recipients receiving induction therapy with rATG or IL2-RA

The outcomes of renal transplantation were compared between groups. There was no significant difference in the incidences of clinically-suspected acute rejection (9.8% vs. 8.8%; *p* = 0.832) and dn-DSA formation (4.9% vs. 5.4%, *P* = 0.44) between the rATG and IL2-RA groups (Table 2). However, the patients in the rATG group had a lower rate of biopsy-proven acute rejection (BPAR, T cell-mediated rejection) as compared with the IL2-RA group (11.1% [1/9] vs. 50% [6/12], *P* = 0.01, Table 2). Among the 7 BPAR cases in this study, 4 patients in IL2-RA group had episode of vascular rejection. The four cases of vascular rejections were treated with single high-dose (2 g/kg) intravenous immunoglobulin (IVIG) treatment; single high-dose (2 g/kg) intravenous immunoglobulin (IVIG) treatment plus a single dose of Rituximab (375 mg/m^2^ body surface area); methylprednisolone 500 mg intravenously for 3 days; and conversion from cyclosporine A to tacrolimus, respectively. Kaplan-Meier curve analysis showed that the graft survival rate was not significantly different between groups (*P* = 0.857, Fig. 2).

**Table 2:** Comparison of the therapeutic outcomes between groups

	rATG *n* = 41	IL2-RA *n* = 147	*P*-value
Clinically-suspected acute rejection, % (n/n)	9.8 (4/41)	8.8 (13/147)	0.832
Graft lost because of rejection, n/n^a^	0/4	4/13	–
Biopsy-proven acute rejection, n/N	1/9	6/12	0.01
Dn-DSA	2 (4.9)	8 (5.4)	0.44

Dn-DSA = de novo donor-specific antigen; IL2-RA = IL-2 receptor antagonist; rATG = rabbit antithymocyte globulin

^a^Denominator is the number of patients with acute rejection

### Cox regression analysis

To further evaluate the impact of induction therapy on the outcomes of renal transplantation, multivariate Cox regression analysis was performed. Three cases in the rATG group and 4 patients in the IL2-RA group were excluded from the Cox regression analysis due to incomplete data. After adjusting for patients’ age, sex, PRA score, HLA mismatch, and the use of corticoids, no difference was found in the incidences of clinically-suspected acute rejection between the IL2-RA group and the rATG group (*P* = 0.757, Table 3).

**Table 3:** Multivariate Cox regression analysis for the independent factors associated with clinically-suspected acute rejection or graft survival

Dependent variable	Covariate	Parameter estimate	Standard error	Risk ratio	*P*-value
Clinically-suspected acute rejection	Sex (male vs. female)	−0.284	0.685	0.753	0.678
	Age, years	−0.028	0.030	0.972	0.341
	HLA mismatch	0.204	0.588	1.227	0.728
	PRA (< 10% vs. > 10%)	0.632	0.923	1.882	0.493
	IL2-RA vs. rATG	−0.152	0.684	0.859	0.824
	Corticoids (no vs. yes)	−13.124	711.226	0.000 ^a^	0.985
Graft survival	Sex (male vs. female)	1.213	1.290	3.362	0.347
	Age, years	0.038	0.059	1.039	0.519
	HLA mismatch	0.613	1.294	1.845	0.636
	PRA (< 10% vs. > 10%)	−11.464	1247.687	0.000 ^a^	0.993
	IL2-RA vs. rATG	−12.260	780.889	0.000 ^a^	0.987
	Corticoids (no vs. yes)	1.873	1.293	6.508	0.148

Multivariate Cox regression analysis was adjusted for patients’ age, sex, PRA score, HLA mismatch, and the use of corticoids

HLA = human leukocyte antigen; IL2-RA = interleukin-2 receptor agonist; PRA = panel reactive antibody; rATG = polyclonal rabbit antithymocyte globulin

^a^, Due to the number of using corticoids was limited, some risk ratios were failed to be estimated and would be represented as 0.000 in SPSS output. However, the *P*-values still indicates there was no significant difference between IL2-RA and rATG groups

When taking graft survival as the dependent variable, multivariate Cox regression analysis showed that there was no significant difference in the graft survival between the IL2-RA group and the rATG group (*P* = 0. 987, Table 3). These results suggested that method of induction therapy had no effect on the incidences of clinically-suspected acute rejection or graft survival.

### Propensity score analysis

Propensity score analysis was performed to investigate the association between independent variables and the choice of induction therapy. As shown in Table 4, it was found that patients with an older age (OR = 0.906, *P* = 0.001) or a higher PRA score (OR = 21.308, *P* < 0.001) were more likely to use rATG as the induction therapy. The probabilities of choosing induction therapy generated by propensity score analysis were further included into Cox regression analysis as a covariate. The results showed that that induction therapy groups (rATG vs. IL2-RA) still had no significant difference in prediction graft survival or clinically-suspected acute rejection (both *P* > 0.05, respectively).

**Table 4:** Propensity score generated by multivariate logistic regression to the choice of induction therapy (rATG or IL2-RA, IL2-RA as reference)

Covariate	Parameter estimate	Standard error	Odds ratio	P-value
Sex (male vs. female)	−0.031	0.609	0.970	0.960
Age (receiver), years	−0.099	0.029	0.906	0.001
HLA mismatch	0.583	0.536	1.792	0.276
PRA (< 10% vs. > 10%)	3.059	0.798	21.308	< 0.001
Age (donor), years	−0.012	0.025	0.988	0.624
Transplantation history (yes vs. no)	2.750	1.366	15.636	0.044

HLA = human leukocyte antigen; IL2-RA = interleukin-2 receptor agonist; PRA = panel reactive antibody; rATG = polyclonal rabbit antithymocyte globulin

### Comparison of the 1-year, 2-year, and 3-year graft survival rate between groups

The 1-year, 2-year, and 3-year graft survival rates were compared between groups. As shown in Table 5, there was no significant difference in the 1-year, 2-year, and 3-year graft survival rate between rATG and IL2-RA groups (all *P* > 0.05), suggesting the comparable outcome between groups.

**Table 5:** Graft survival rates at year 1, 2, and 3

Graft survival rate	rATG	IL2-RA	*P*
Year 1	37 (100.0)	123 (97.6)	1.000
Year 2	21 (100.0)	73 (96.1)	1.000
Year 3	10 (100.0)	44 (93.6)	1.000

### Complications after renal transplantation

As shown in Table 6, the incidences of complications in both treatment groups were all comparable, including total infection, pneumonia, liver injury and myelosuppression (all *P* > 0.05), indicating that method of induction therapy had no effect on the incidences of post-transplantation complications.

**Table 6:** Comparison of the incidences of post-transplantation complications between groups

Event, *n* (%)	rATG *n* = 41	IL2-RA *n* = 147	*P*-value
Liver injury	2 (4.9)	15 (10.2)	0.31
Myelosuppression	0 (0)	3 (2.0)	0.36
Infection	8 (19.5)	24 (16.3)	0.96
Pneumonia	8 (19.5)	24 (16.3)	0.82

IL2-RA = Interleukin-2 receptor antagonist; rATG = rabbit antithymocyte globulin

Moreover, a multivariate logistic regression analysis was performed to investigate the independent variables associated with overall infection. As indicated in Table 7, no significance was identified. Again, these results suggested the comparable outcome between groups.

**Table 7:** Multivariate logistic regression analysis for the independent factors associated with overall infection

Dependent variable	Covariate	Parameter estimate	Standard error	Odds ratio	*P*-value
Infection (overall)	Sex (male vs. female)	−0.803	0.690	0.448	0.244
	Age, years	−0.003	0.025	0.997	0.920
	HLA mismatch	−0.771	0.659	0.463	0.242
	PRA (< 10% vs. > 10%)	−0.013	0.950	0.987	0.989
	IL2-RA vs. rATG	0.512	0.600	1.669	0.393
	Corticoids (no vs. yes)	0.748	0.851	2.114	0.379

Multivariate Cox regression analysis was adjusted for patients’ age, sex, PRA score, HLA mismatch, and the use of corticoids

HLA = human leukocyte antigen; IL2-RA = interleukin-2 receptor agonist; PRA = panel reactive antibody; rATG = polyclonal rabbit antithymocyte globulin

## Discussion

In this study, we compared the therapeutic efficacy and safety between IL2-RA and rATG induction therapies in Chinese recipients of living donor kidney transplantation. The results demonstrated that the rATG group had a higher PRA score and more cases with complete HLA mismatches than IL2-RA group, suggesting a higher immunological risk. The incidences of clinically-suspected acute rejection and dn-DSA were not significantly different between the rATG and IL2-RA groups, but rATG group had a lower rate of BPAR than the IL2-RA group, when diagnostic biopsy was performed. Kaplan-Meier curve analysis showed that the graft survivals of two groups were comparable. In addition, the multivariate Cox regression analysis demonstrated that the method of induction therapy was not an influential factor associated with either clinically-suspected acute rejection or graft survival. The 1-year, 2-year, and 3-year graft survival rates were comparable between groups. Furthermore, the incidences of complications (infections, pneumonia, liver injury and myelosuppression) were all comparable between groups. Taken together, these results suggested that rATG could be a safe and efficient immunosuppressant and when used in a Chinese recipient population with a higher immunological risk in living donor renal transplantation.

Previous studies have demonstrated that patients receiving rATG induction therapy have an acute rejection rate of around 10% [6, 9–13]. A multiple-center study by US transplant centers based on the data from 1816 patients in the Thymoglobulin Antibody Immunosuppression in Living Donor Recipients (TAILOR) Registry reported that the BPAR is 8.3% at 12 months post-transplantation [10]. Likewise, a retrospective cohort analysis of the Organ Procurement and Transplantation Network (OPTN) registry by Tanriover et al. have reported similar 1-year acute rejection rates (either biopsy-confirmed or clinically treated) of patients receiving rATG induction therapy with (9.6%, *n* = 8552) or without (9.0%, *n* = 4905) steroid maintenance therapy [9]. In this study, the rATG group had a rate of clinically-suspected acute rejection of 9.8%, which is consistent with the above reports. On the other hand, the IL2-RA group in this study had a clinically-suspected acute rejection rate of 8.8%, which is slightly lower than rates of acute rejection in Tanriover et al.’s study (11.7% in patient with steroid maintenance and 10.5% in those without steroid maintenance at 12 months) [9].

Our results showed that rATG group had a higher PRA score and more complete HLA mismatches than the IL2-RA group, indicating a higher immunological risk in the rATG group. However, after adjusting for the baseline confounders including PRA score and complete HLA mismatch, our multivariate Cox regression model still demonstrated that there were no differences in either the risk of clinically-suspected acute rejection or graft survival between the IL2-RA group and the rATG group. This finding is in line with a prospective study including 213 cases of living-donor renal transplantation by Huang et al. [14]. Their study reports that there are no significant differences in the acute rejection rates, DGF rates, graft loss and death between the IL2-RA group and the rATG group [14].

The 2009 KDOQI guideline suggests that IL2-RA induction therapy could reduce the rate of infection in renal transplantation as compared with the rATG induction therapy [2]. Consistently, the prospective study in living-donor renal transplantation by Huang et al. have reported that the rATG group has a significantly higher infection rate than the IL2-RA group (85.8% vs. 75.2%, *P* = 0.03) [14]. In contradiction to their observation, our result revealed that the post-transplantation infection rate was similar between the two treatment groups. This discrepancy may be attributed to the total cumulative dose of rATG is lower in our study (3 mg/kg) than in Huang et al.’s study (5 mg/kg) [14]. The rATG dose used in this study is the standard dose typically used in Chinese kidney transplant patients, with the purpose to reduce infectious complication [14–16]. Moreover, although total cumulative dose of prophylactic intravenous ganciclovir within the first 2 weeks is lower in our study, however, the daily dose of oral ganciclovir in the following 90 days in our study (3 g/day) is twice as high as that in Huang et al.’s study (1.5 g/day) [14]. This may also contribute to the decrease in infection rate. rATG is a more potent immunosuppressant and could induce a higher risk of infection than IL2-AR [17]. However, our study showed that the incidence of infectious complication was lower in the rATG group than in the IL-2AR group, which may be attributed to the relatively young age of recipients in the rATG group or relatively small sample size of this study. Taken together, our observations indicated that rATG induction therapies in recipients of living donor kidney transplantation could simultaneously achieve low rates of post-transplantation infection and acute rejection, especially for the recipients with high immunological risk.

De novo formation of DSAs directed against HLA has been recognized as one of the major risk factors for allograft failure [18–20]. In this study, the incidence of dn-DSA formation was comparable between the rATG and the IL2-RA groups (4.9% vs. 5.4%). A review article including 12 studies by Lionaki et al. has shown that the incidence rates of dn-DSA formation in kidney transplant recipients range from 5.5 to 32% [19]. The marked variation of incidence rates of dn-DSA formation among studies is due to the diversity of methods used for antibody detection [19]. Among the 12 reviewed studies, only 3 ones report the rate of dn-DSA lower than 10% [19]. Hence, the incidence of dn-DSA formation in this study was relatively low. However, Everly et al. have reported that 47 out of 189 (25%) patients developed dn-DSA within 10 years post-transplantation. Among them, there are 42.6, 36.2 and 21.3% of patients developing dn-DSA antibody within the first year, between 1 and 5 years and after 5 years post-transplantation, respectively [21]. Therefore, given the lack of long-term follow-up data in the present study, the rates of dn-DSA may have been under-estimated and a long-term follow-up is necessary.

There are several limitations of this study. Due to the retrospective nature, some patients had missing or incomplete data, resulting in inaccuracy of the data (such the date of acute rejection and graft survival outcome) which could affect the analysis results to some extent. However, multivariate Cox regression analysis adjusted for baseline characteristics was conducted to eliminate the confounding factors. In addition, the lack of systematic diagnostic biopsy for rejection is a major drawback of this study, since it has been suggested that a transplant renal biopsy should be carried out before treating an acute rejection episode [22]. The incidence of vascular rejection in the BPAR cases was high (66.7%,, 4/6), which may be due to the fact that our previous biopsies strategy was indication biopsies, in which biopsies was performed only when the patient had clinical symptoms and the treatment was not effective. This was a limitation. At present, our biopsies strategy has been improved to surveillance biopsies. In this study, the method of induction therapy was determined by the attending physician based on the immunological and infection risks of each patient. Nevertheless, the risk may be overestimated or underestimated in some patients, resulting in an inadequate induction therapy. This bias may interfere with the analysis results of this study. Therefore, a well-design prospective study with a large sample size is necessary to further validate the findings of this study. All these limitations should be addressed in future studies.

## Conclusions

In summary, our findings demonstrated that rATG could be a safe and efficient immunosuppressant when used in a Chinese recipient population with a higher immunological risk in living donor renal transplantation.

## Abbreviations

ALT: Alanine transaminase
AST: Aspartate transaminase
BPAR: Biopsy-proven acute rejection
CMV: Cytomegalovirus
DSA: Donor-specific antibody
HLA: Human leukocyte antigen
IL2: Interleukin-2
OPTN: Organ Procurement and Transplantation Network
PCP: Pneumocystis carinii pneumonia
SD: Standard deviation
